# Histopathological Changes in Laparoscopic Sleeve Gastrectomy Specimens: Prevalence, Risk Factors, and Value of Routine Histopathologic Examination

**DOI:** 10.1007/s11695-016-2525-1

**Published:** 2017-01-06

**Authors:** Tamer Safaan, Moataz Bashah, Walid El Ansari, Mohsen Karam

**Affiliations:** 1 Department of General Surgery, Hamad General Hospital, Hamad Medical Corporation, Doha, State of Qatar; 2 Department of Bariatric Surgery, Hamad General Hospital, Hamad Medical Corporation, Doha, State of Qatar; 3 Department of Surgery, Hamad General Hospital, Hamad Medical Corporation, Doha, State of Qatar

**Keywords:** Morbid obesity, Sleeve gastrectomy, Stomach, Histopathologic examination, *H*. *pylori*, GIST, Intestinal metaplasia

## Abstract

**Background:**

Laparoscopic sleeve gastrectomy (LSG) is a common surgical therapeutic option for obese patients, with debate about the value of routine histopathologic examination of LSG specimens. We assessed the following: prevalence of different histopathologic changes in LSG specimens, risk factors associated with premalignant and with frequent histopathologic changes, and whether routine histopathologic examination is warranted for LSG patients with nonsignificant clinical history.

**Methods:**

Retrospective review of records of all LSG patients operated upon at Hamad General Hospital, Qatar (February 2011–July 2014, *n* = 1555), was conducted. Risk factors (age, BMI, gender, and *Helicobacter pylori*) were assessed in relation to specific abnormal histopathologic changes.

**Results:**

Mean age and BMI of our sample were 35.5 years and 46.8, respectively. Females comprised 69.7% of the sample. Normal histopathologic specimens comprised 52% of the sample. The most common histopathologic changes were chronic inactive gastritis (33%), chronic active gastritis (6.8%), follicular gastritis (2.7%), and lymphoid aggregates (2.2%). We observed rare histopathology in 3.3% of the sample [e.g., intestinal metaplasia and gastrointestinal stromal tumor (GIST)]. Older age was associated with GIST and intestinal metaplasia (*P* = 0.001 for both). Females were associated with chronic active gastritis (*P* = 0.003). *H. pylori* infection was associated with follicular gastritis, lymphoid aggregates, GIST, intestinal metaplasia, and chronic active gastritis (*P* < 0.001 for each).

**Conclusion:**

Older age, *H. pylori*, and female gender are risk factors for several abnormal histopathologic changes. Histopathologic examination of LSG specimens might harbor significant findings; however, routine histopathologic examination of all LSG specimens, particularly in the absence of suggestive clinical symptoms, is questionable. The association between female gender and chronic active gastritis; and the association between H. pylori infection and GIST are both novel findings that have not been previously reported in the published literature.

## Introduction

Obesity is a serious health problem worldwide where >1.9 billion adults are overweight, of which 600 million are obese [1]. In the Middle East, 74–86% of women and 69–77% of men are either obese (BMI ≥ 30) or overweight (BMI 25–29.9) [2], with increased risk of type 2 diabetes, hypertension, hyperlipidemia, coronary artery disease, and shorter life span [3, 4]. Nonsurgical treatment of obesity (physical activity, diet/behavior modification, and pharmacotherapy) seems ineffective with severely obese patients (BMI > 40), or those with BMI 35–39.9 combined with comorbidities. Such patients can undergo either primarily restrictive (e.g., laparoscopic sleeve gastrectomy—LSG) or malabsorptive surgery (e.g., Roux-en-Y gastric bypass) [5]. While LSG is now a common procedure, however, the published literature suggests several shortcomings.

First, most research explored the clinical aspects, surgical techniques, and postoperative complications of LSG [6], with few studies examining the histopathologic outcomes, resulting in a scarcity of data about gastric histopathologic changes in LSG patients [7]. Second, while LSG patients may be presumed to have no significant gastric pathology, the literature is highly inconsistent as whether this is usually the case. In the USA, 8.4% of cases had unforeseen findings necessitating clinical follow-up [8]; in Kuwait, no normal specimens were reported, and 74.4% of the 656 LSG specimens had element/s of chronic gastritis [9]; and in New Zealand, >50% of the LSG specimens demonstrated histopathologic abnormality [10]. Conversely, others found that most post LSGs had no pathologic alteration, that a minority had significant pathologic findings [7, 11], and that routine microscopic examination of LSG specimens was unnecessary [6].

In addition, there is a paucity of research on gastric histopathologic changes of morbidly obese LSG patients across the eastern Mediterranean countries, except for Saudi Arabia [12], Kuwait [9], and United Arab Emirates [6]. Furthermore, most of the scarce published literature on histopathologic changes in morbidly obese LSG patients investigated modest sample sizes, e.g., 87 patients [13], 145 patients [7], or 248 patients [8], with the larger studies comprising 310 [11] or 656 patients [9]. Finally, while some risk factors (e.g., gender, age, and *Helicobacter pylori* infection) seem associated with specific abnormal histopathologic changes in general populations [14–23], these risk factors have not been examined across samples of LSG patients. Unsurprisingly, there still remains much debate as to whether routine histopathologic examination of LSG specimens is required, with support [9] or nonsupport [6] for such routine examination.

Given the unequivocal opinion about the following: (a) whether abnormal gastric histopathologic changes are evident in high percentages of morbidly obese LSG patients, (b) the risk factors associated with abnormal gastric histopathologic changes across LSG patients, and (c) the value of routine gastric histopathologic examination of LSG specimens, the current study examined 1555 LSG specimens at Hamad General Hospital in Doha, the largest hospital in the State of Qatar. The specific objectives were to assess, in post LSG specimens:

1. The types of abnormal histopathologic changes and their prevalence
2. Risk factors associated with potentially *premalignant* abnormal histopathologic change, e.g., follicular gastritis, lymphoid aggregates, mucosa-associated lymphoid tissue lymphoma (MALT lymphoma), GIST (gastrointestinal stromal tumor), and intestinal metaplasia [24–26]
3. Risk factors associated with a particularly *frequent* abnormal histopathologic change (active chronic gastritis)
4. Whether routine histopathologic examination of LSG specimens is justified in patients with nonsignificant clinical history

This is the first study in Qatar, and to the best of our knowledge, the current study could be the largest of its kind globally to examine the types of gastric histopathologic changes in LSG patients, to explore the risk factors associated with specific potentially premalignant and/or particularly frequent LSG histopathologies, and to assess whether routine histopathologic examination of LSG specimens is justified in patients with nonsignificant clinical history. Only one previous study [6] had previously investigated the role of routine microscopic examination of LSG specimens, albeit employing a sample size (*n* = 546) that was roughly one third the number of the patients included in our sample. Hence, we present the largest (*n* = 1555) series in the published literature. Given that LSG is rapidly developing as a main bariatric operation [7], such considerations highlight the importance of the current study and the significance of its findings in contributing to the evidence base.

## Materials and Methods

### Ethics and Sample

The current study was undertaken in Qatar at Hamad General Hospital (HGH) in Doha which is part of Hamad Medical Corporation (HMC, equivalent of Ministry of Health). The Medical Research Centre at Hamad Medical Corporation approved the study protocol (Proposal No. 16202/16). We retrospectively retrieved and systematically reviewed the demographic, clinical, and histopathologic data extracted from the medical records of all patients who had undergone primary LSG for morbid obesity at HGH from February 2011 to July 2014 (*n* = 1555). The clinical findings and postoperative course of patients were also noted.

### Procedures and Data Collection

As a standard protocol at HMC, all LSG patients undertook a routine preoperative esophagogastroduodenoscopy (OGD) and *Campylobacter*-like organism (CLO) test to assess their *H. pylori* infection status. All CLO-positive patients received standard triple therapy that consists of amoxicillin and clarithromycin for 2 weeks and proton pump inhibitor (PPI) for 2 months. All gastric specimens of LSG patients were examined by our histopathology department macro- and microscopically, and a diagnosis was provided. Clinical data about postoperative follow-up for LSG patients with significant gastric pathology were retrieved from the electronic medical records, and these cases were discussed with the appropriate consultant who was managing the patient.

### Statistical Analysis

SPSS 22.0 (SPSS Inc., Chicago, IL), with significance level set at *P* < 0.05, was used for the statistical analyses of data from 1555 LSG. The types of abnormal histopathologic changes and their prevalence were computed. Descriptive statistics (frequency and percentage, and mean ± SD with median and range) summarized participants’ demographic and other clinical characteristics.

We conducted comparisons to assess the risk factors associated with each of: potentially *premalignant* abnormal histopathology [follicular gastritis and lymphoid aggregates (precursors of MALT), GIST, intestinal metaplasia]; and, frequent abnormal histopathology (chronic active gastritis). The variables included in the comparisons comprised age, gender, preoperative BMI, and the presence/absence of *H*. *pylori* infection. Chi-square (χ^2^) / Fisher exact test as appropriate assessed any associations between two or more categorical variables. Unpaired *t* test or Mann-Whitney *U* test as applicable examined any associations between two independent groups of quantitative variables.

## Results

### Characteristics of the Sample

Table 1 depicts the sample’s demographic information. Females comprised 69.7% of the sample. Mean ages for males and females were similar (overall mean age = 35.5 ± 10.7 years), while males had slightly higher BMI than females (overall mean BMI = 46.8 ± 8.4).

**Table 1:** Characteristics of 1555 LSG patients

Gender	*n* (%)	Age		BMI (*M ±* SD)
		*M* ± SD	Range	
Male	471 (30.3)	35.3 ± 11.4	13–74	48 ± 9.1
Female	1084 (69.7)	36 ± 10.3	14–65	46.3 ± 8.1

*M* mean, *SD* standard deviation

### Types and Prevalence of LSG Histopathologic Changes

Table 2 shows the diversity of diagnoses of the LSG histopathologic specimens. Slightly more than half (52%) of the specimens were normal. The first most common histopathologic change (33%) was chronic inactive gastritis (categorized into mild/moderate according to the amount of chronic inflammatory cells in the lamina propria). The second most common histopathologic change (6.8%) was active chronic gastritis (chronic inflammatory cells + polymorphonuclear lymphocytes in the lamina propria). To a lesser extent, follicular gastritis (accumulation of plasma cells and lymphocytes with germinal centers) comprised 2.7% of cases, and lymphoid aggregates (accumulation of plasma cells and lymphocytes without germinal centers in the lamina propria) were evident among 2.2% of the specimens.

**Table 2:** Types and prevalence of LSG histopathologic specimens

Histopathology of LSG specimen	*n* (%)
Normal (no specific histopathologic change)	810 (52)
Abnormal (specific histopathologic change evident)	745 (48)
Chronic inactive gastritis (mild or moderate)	512 (33)
Chronic active gastritis	105 (6.8)
Follicular gastritis	43 (2.7)
Lymphoid aggregate	35 (2.2)
Intestinal metaplasia	22 (1.4)
GIST	11 (0.7)
Fundic gland polyp	3 (0.19)
Atrophic chronic gastritis	3 (0.19)
Lymphplasmacytic noncaseating granuloma	2 (0.13)
Submucosal fibrosis with eosinophil-rich chronic inflammation	1 (0.06)
Focal gangrenous necrosis	1 (0.06)
Autoimmune gastritis	1 (0.06)
Mesothelial chronic inflammation	1 (0.06)
Focal prominence of intramural neural tissue	1 (0.06)
Dysplastic neuroendocrine nodule	1 (0.06)
Leiomyoma	1 (0.06)
Pancreatic heterotopia	1 (0.06)
Gastric lipoma	1 (0.06)

*LSG* laparoscopic sleeve gastrectomy, *GIST* gastrointestinal stromal tumor

We also observed several of the rarer diagnoses: three cases of benign fundic gland polyp, one gastric lipoma, one pancreatic heterotopia, and one focal prominence of the intramural neural tissue. In addition, a very low proportion (<2.5%) of our patients had incidental clinical diagnoses that usually require close follow-up (e.g., leiomyoma, intestinal metaplasia, GIST, and dysplastic neuroendocrine nodule); however, none of these diagnoses exhibited any clinical significance in terms of postoperative complications, and these patients had smooth postoperative course.

### Risk Factors Associated with Potentially Premalignant Abnormal Histopathologic Change

These potentially premalignant abnormal histopathologic changes included the following: (a) precursors of malignancies, e.g., follicular gastritis and lymphoid aggregates, as both can be predecessors of gastric MALT lymphoma; (b) benign tumor with potential for malignancy (GIST); and (c) precursors for gastric adenocarcinoma (e.g., intestinal metaplasia). We compared these abnormal histopathologic changes in terms of patient’s age, gender, BMI, and associated *H. pylori* infection.

Table 3 depicts that the comparison between cases of follicular gastritis and lymphoid aggregate specimens collectively (precursors of MALT) and normal specimens (controls) revealed no differences between the two groups in terms of age, BMI, or gender. However, *H. pylori* infection was significantly more associated with follicular gastritis and with lymphoid aggregates compared with normal specimens.

**Table 3:** Patients with follicular gastritis or lymphoid aggregates compared with normal specimens

	Follicular gastritis or lymphoid aggregates (*n* = 78)	Normal specimens (*n* = 90)	*P*
Age (*M*, SD)	35 (9.4)	35.3 (8.5)	0.848
BMI (*M*, SD)	46.7 (9.5)	47.2 (8.1)	0.708
Gender (*n*, %)			0.39
Male	23 (29.5)	25 (27.8)	
Female	55 (70.5)	65 (72.2)	
*H*. *pylori* (*n*, %)			<0.001
Positive	63 (80.8)	3 (3.3)	
Negative	15 (19.2)	87 (96.7)	

*M* mean, *SD* standard deviation

A more detailed comparative sub-analysis between the individual cases of follicular gastritis, lymphoid aggregates, and normal specimens was undertaken (Table 4). While follicular gastritis was present in the older and more obese patients as compared with lymphoid aggregates, the differences were not significant. However, *H. pylori* infection was significantly more associated with each of follicular gastritis and with lymphoid aggregates, compared to normal specimens (*P* < 0.0001).

**Table 4:** Follicular gastritis patients compared with lymphoid aggregates and with normal specimens

	Follicular gastritis (*n* = 43)	Lymphoid aggregates (*n* = 35)	Normal specimens (*n* = 90)	*P*
Age (*M*, SD)	37.1 ± 9.4	32.4 ± 8.7	35.2 ± 8.5	0.067
BMI (*M*, SD)	48.3 ± 10.9	44.8 ± 7.2	47.2 ± 8	0.209
Gender (*n*, %)				0.39
Male	10 (23.3)	13 (37.1)	25 (27.8)	
Female	33 (76.7)	22 (62.9)	65 (72.2)	
*H*. *pylori* (*n*, %)				<0.0001
Positive	37 (86)	26 (74.3)	3 (3.3)	
Negative	6 (14)	9 (25.7)	87 (96.7)	

*M* mean, *SD* standard deviation

Table 5 depicts the comparisons between patients with GIST, intestinal metaplasia (precursor of gastric adenocarcinoma), and normal specimens. Older age was significantly associated with GIST and intestinal metaplasia when compared to normal specimens; and GIST patients were slightly older than those with intestinal metaplasia (49.4 vs. 47.4 years). While both GIST and intestinal metaplasia were more among females, these associations did not reach statistical significance. Both histopathologic changes were significantly associated with *H. pylori* infection, with intestinal metaplasia exhibiting a stronger association than GIST.

**Table 5:** GIST patients compared with intestinal metaplasia and with normal specimens

	GIST (*n* = 11)	Intestinal metaplasia (*n* = 22)	Normal specimens (*n* = 42)	*P*
Age (*M*, SD)	49.4 ± 10.4	47.4 ± 11.1	35 ± 13.9	0.0001
BMI (*M*, SD)	43.2 ± 8.1	44.5 ± 8.6	46.1 ± 8	0.546
Gender (*n*, %)				0.713
Male	2 (18.2)	4 (18.2)	11 (26.2)	
Female	9 (81.8)	18 (81.8)	31 (73.8)	
*H*. *pylori* (*n*, %)				0.0001
Positive	8 (72.2)	20 (90.9)	2 (4.8)	
Negative	3 (27.3)	2 (9.1)	40 (95.2)	

*GIST* gastrointestinal stromal tumor

### Risk Factors Associated with a Particularly Frequent Abnormal Histopathologic Change

A particularly frequent abnormal histopathologic change is chronic active gastritis. It is a frequent precursor of peptic ulcer. Table 6 shows the comparison of patients with chronic active gastritis (*n* = 105) vis-a-vis normal specimens (*n* = 109). Although there were no significant differences between both groups in terms of age and BMI, active chronic gastritis patients were significantly more likely to be females and to have *H. pylori* infection.

**Table 6:** Chronic active gastritis patients compared with normal specimens

	Chronic active gastritis (*n* = 105)	Normal specimens (*n* = 109)	*P*
Age (*M*, SD)	38.4 (11.3)	39.5 (6.3)	0.37
BMI (*M*, SD)	47.7 (7.6)	47.1 (7.7)	0.59
Gender (*n*, %)			0.003
Male	35 (33.3)	58 (53.2)	
Female	70 (66.7)	51 (46.8)	
*H*. *pylori* (*n*, %)			<0.001
Positive	67 (63.8)	36 (33)	
Negative	38 (36.2)	73 (67)	

*M* mean, *SD* standard deviation

## Discussion

Our sample comprised gastric specimens from 1555 patients (*M*_age_ = 35.5 years, females = 69.7%). Our mean age and gender composition were in agreement with others (AbdullGaffar et al. 2016, *M*_age_ = 33, females = 64.2%; Raess et al. 2015, females = 69.2%; Almazeedi et al. 2013, *M*_age_ = 33.6, females = 73.2%) [6, 8, 9]. In support of the current study, other research [6, 8, 9] has also suggested that obesity was more prevalent in females. Our mean BMI was 46.8 for the whole sample and was higher for males (48) than females (46.3).

In terms of normal histopathology and abnormal histopathologic changes, Table 7 summarizes the comparisons of our findings with other research. As for the normal histopathology among LSG patients and their prevalence, slightly more than half (52%) of our LSG specimens were normal, almost equal to those reported in the USA (Clapp et al. 2015, 50.3%) [7] and UAE (54%) [6], but slightly higher than in the USA (Raess et al. 2015) [8] or New Zealand [10], where there were no specific pathologic changes in 35.2 and 46.3% of LSG patients, respectively. Nevertheless, our 52% normal LSG specimens contrasted with Kuwait (no normal specimens among post LSG patients) [9] and was lower than in the USA (Ohanessian et al. 2016, 69% normal specimens) [11]. Indeed, in agreement with the current study, across the five previously mentioned studies [6–8, 10, 11], the most common diagnosis was normal specimen. Collectively, such findings might suggest a limited value of routine histopathologic examination among LSG patients.

**Table 7:** Comparison of prevalence of histopathologic findings of LSG specimens in different studies and whether preoperative OGD was undertaken

	Histopathology specimens							Preoperative OGD undertaken
	Normal	Chronic inactive gastritis/chronic gastritis	Chronic active gastritis	Follicular gastritis and/or lymphoid aggregates	Intestinal metaplasia	GIST	*H*. *pylori*	
Current study	52	33	6.8	4.9	1.4	0.7	40.9	All patients
Almazeedi et al. (2013) [9]	0	74.4	7.5	14.5^a^	0.2	0.2	7.3	All patients
Clapp et al. (2015) [7]	50.3	44.1	^c^	4.1	^c^	^c^	18	Selected patients
AbdullGaffar et al. (2016) [6]	54	45	8.4	^c^	0.7	^c^	10	All patients
Raess et al. (2015) [8]	35.2	24	^b^	31.2	2	^c^	5.2	Selected patients
Lauti et al. (2016) [10]	46.3	38.9	^c^	^c^	2.6	0.4	8.6	Selected patients
Ohanessian (2015) [11]	69	13	1.6	^c^	1.3	1	3.2	Selected patients

All cells represent percentages

*GIST* gastrointestinal stromal tumor

^a^>1 diagnoses added together

^b^The given diagnosis is not reported as it has been probably reported collectively in diagnosis a

^c^The given diagnosis was not reported

As for the common abnormal histopathologies, our four most common abnormal histopathologies were chronic inactive gastritis (33%) and chronic active gastritis (6.8%), while follicular gastritis and lymphoid aggregates comprised 2.7% and 2.2% of our specimens, respectively. Our first most common abnormal histopathology (chronic inactive gastritis, 33%) is in agreement with the UAE (45%) [6]. Almazeedi et al.’s [9] four most common abnormal histopathologies were chronic gastritis (74.4% of total specimens), chronic active gastritis (7.5%), follicular gastritis (9.6%), and active follicular gastritis (4.9%). Our findings are in partial agreement with Almazeedi et al. [9]. However, direct comparisons of our findings with Almazeedi et al. [9] and with others were hindered by the different classifications of abnormal histopathological changes that were employed in different countries. For instance, while our histopathology laboratory categorized follicular gastritis in 2.7% of our specimens with no distinction between follicular and active follicular gastritis, Almazeedi et al. [9] described two subtypes: follicular and active follicular gastritis (9.6 and 4.9%, respectively). Likewise, the four most common abnormal histopathologies that Raess et al. [8] found were lymphoid aggregates (31.2%), gastritis (all subtypes, 12%), chronic inflammation (12%), and fundic gland polyps (7.6%). Our lymphoid aggregate rate (2.2%) was much lower than that of Raess et al. [8]. Again, the different abnormal histopathological classifications employed by different researchers in different countries rendered the accurate and direct appraisals of findings across studies/countries difficult. However, applying Raess’s [8] criteria on our specimens would have resulted in 39.8% chronic gastritis and chronic inflammation, as contrasted to the 24% that they reported. Such findings suggest the urgent need to a more unified and standardized system of classification of abnormal histopathologies.

As for other abnormal histopathologies, Table 7 shows that our 1.4% intestinal metaplasia prevalence was very similar to that of Ohanessian et al. (2016) (1.3%) [11], while being either lower or higher than other research. Likewise, our GIST prevalence was lower than that Ohanessian et al. (2016) (1%) [11].

Certainly, the discrepancies in histopathological classifications employed by different researchers posed challenges for precise and relevant comparisons of findings across countries. Such discrepancies materialized into four different forms. First, some authors grouped different diagnoses as one category, but we classified them separately (individual diagnoses), e.g., Raess et al. [8] compiled all subtypes of gastritis as one category (12%), while we differentiated gastritis into chronic inactive gastritis (33%, whether mild/moderate) and chronic active gastritis (6.8%). Secondly and conversely, some authors classified diagnoses separately when we grouped them as one category, e.g., Almazeedi et al. [9] made a discrepancy between follicular gastritis and active follicular gastritis (both accounted for 14.5%), while our histopathology laboratory did not undertake a similar discrepancy. Thirdly, to the best of our knowledge, some authors did not clarify whether certain diagnoses were included in a given category, e.g., Clappet al. [7] and Lauti et al. [10] reported 44.1 and 38.9% chronic gastritis, respectively; however, it was not entirely clear whether they meant chronic active, inactive gastritis, or alternatively both, as they did not explicitly report on this. Fourthly, some authors did not report at all on some diagnoses, e.g., follicular gastritis and/or lymphoid aggregates were not reported by others [6, 10, 11], without clarity as to whether such lack of reporting follicular gastritis/lymphoid aggregates was due to complete absence of such diagnoses among their specimens or otherwise (e.g., not the primary focus of the study or selective reporting) (Table 7). Future research would benefit from exploring the implications of the following: (a) the different histopathological classifications employed in different countries and work towards a more unifying and standardized classification; and, (b) mandatory reporting on all the categories of such a unified system. Such actions would assist in precise and meaningful comparisons across different studies/countries and contribute to a solid evidence base useful for practice.

In terms of associated risk factors, the current study assessed age, BMI, gender, and *H. pylori* infection as risk factors for abnormal histopathologies (particularly chronic active gastritis, lymphoid aggregates and follicular gastritis, GIST, and intestinal metaplasia). We observed that BMI and gender were not associated with any of these abnormal histopathologies, except for chronic active gastritis which was significantly associated with females. To the best of our knowledge, the current study could be the first to report such an association between chronic active gastritis and gender. Others [27] have suggested that obesity may be a risk factor for gastritis; and as obesity is more prevalent in females, such considerations might contribute to explain our findings of the significant association between chronic active gastritis and female gender.

As for the other risk factors under examination, we observed several important findings. First, in terms of age, we found that GIST was more associated with older age (*M*_age_ 49.4 ± 10.4, *P* = 0.0001). Such an association is supported by the literature which showed that GIST occurs predominantly in middle-aged and older individuals and seldom under the age of 40. For instance, in the USA, mean age of GIST patients at time of diagnosis was 63 [14]. In addition, we noted that older age was significantly associated with intestinal metaplasia (*P* = 0.0001) which is in agreement with the literature that intestinal metaplasia is more prevalent among the advanced age groups [16].

As regards to *H. pylori* infection as a risk factor, it was diagnosed in 40.9% of our LSG specimens, much higher than in USA, Kuwait, and New Zealand (5.2%; 7.3%; and 8.6%, respectively) [8–10]. Such high prevalence of this bacterium in our study might suggest the high prevalence in our general community. Indeed, studies have confirmed high prevalence of *H. pylori* (between 20% and 97%) in Middle Eastern populations, e.g., Iran, Egypt, Libya, Saudi Arabia, and Turkey [28]. This is important, as *H. pylori* is responsible for worldwide chronic bacterial infection affecting about half of the world’s population; is associated with morbidity/mortality; is a risk factor for gastritis, duodenitis, peptic ulcer, and other benign and malignant diseases; has direct/indirect impacts on economic and general well-being of patients; and its eradication is challenging [18, 19, 28]. Nevertheless, in contrast, while reports have suggested that *H. pylori* could be a cause for gastritis, others found a moderate prevalence of gastritis among adolescents undergoing LSG, but only a small number of these patients were *H. pylori* positive [29]. Our *H. pylori* infection was significantly associated with chronic active gastritis (*P* = <0.001), with follicular gastritis and with lymphoid aggregates (*P* = <0.0001), with intestinal metaplasia and also surprisingly with GIST (*P* = 0.001). Previous studies have similarly showed associations of *H. pylori* with such conditions [16, 20, 22, 23]. However, in terms of the association of *H. pylori* with GIST, with the exception of a study reporting the strong association between GIST and *H. pylori* [30], to the best of our knowledge, there does not seem to be research to date that explored the association between *H. pylori* infection and GIST. Future inquiries could benefit from confirming or refuting such an association between *H. pylori* infection and GIST, particularly that most GISTs could have malignant potentials [31].

There still remain controversies about the value of routine OGD prior to bariatric surgery. We undertook routine preoperative OGD for all our LSG patients, in accordance with the European guidelines (EAES) [32]. However, the Society of American Gastrointestinal and Endoscopic Surgeons (SAGES) recommended that OGD “may be used if suspicion of gastric pathology exists” before bariatric surgery [33]. Yet, others [6] undertook preoperative OGD for all LSG patients, and *H. pylori* testing was done only in symptomatic patients; and Almazeedi et al. [9] undertook preoperative OGD and CLO testing for all LSG patients, and where CLO testing was positive, then triple therapy was given. Nevertheless, other researchers [8] conducted preoperative OGD in <5% of cases, only where esophageal web, duodenal ulcer, or mass lesion were identified. Some studies [7] undertook preoperative OGD in selected patients, based on patient’s symptoms, while other researchers [10, 11] respectively conducted preoperative OGD in only 21 out of 976 patients and 8 out of 310 patients. Whereas assessing the role of preoperative OGD in LSG is beyond the scope of the current study, such inconsistencies strongly suggest that more research of the role of preoperative OGD in LSG is urgently required, as there remains much debate about whether OGD prior to bariatric surgery should be routine or selective [34].

Finally, we observed no malignancies amongst our abnormal histopathologic specimens, in agreement with all the studies cited in Table 7 that confirmed no malignancies in post LSG specimens. We completely resected all the benign tumors (e.g., GIST) that we found, and patients had a smooth postoperative recovery with no complications. Our observation of such lack of malignancies further raises the question of any added value of routine histopathologic examination of post LSG specimens, as others have rightly noted [6].

Certainly, parallel evidence for routine histopathologic examination among other conditions across surgical specialties, e.g., routine examination of tonsils, appendix, gallbladder, and hemorrhoid specimens has suggested the paucity of incidental histopathologic findings pertinent to patients’ management, particularly in the absence of intraoperative or gross abnormalities, a point that proposes that routine histologic analysis may be omitted [35, 36]. Indeed, in the current atmosphere of financial accountability, cost-effectiveness of hospital resources, and evidence-based decision-making, routine histopathological examination of certain surgical specimens has been repeatedly questioned [36–39]. Hence, careful and cautious selection premised on patients’ clinical picture and operative findings of any suspicious lesion/s would appear to be more cost-effective.

A limitation of this study is that we undertook preoperative esophagogastroduodenoscopy routinely, in line with others [9], and patients with positive *H*. *pylori* had triple therapy prior to surgery. Such undertaking may have influenced the frequencies of histopathological abnormalities identified in the LSG specimens. In addition, although the hospital (HGH) where the current study was undertaken is the largest hospital in Qatar and captures the great majority of the LSGs, however, there also exist a few smaller private hospitals in Qatar that do undertake LSG, and information about their findings would have been beneficial.

## Conclusion

To our knowledge, this is the largest study of histopathological diagnoses in LSG patients. A total of 52% of our specimens were normal. The most common abnormal histopathological change was chronic inactive gastritis (33%). Some rare (3.3%) benign lesions were found including intestinal metaplasia, GIST, gastric lipoma, and leiomyoma. Older age was associated with GIST and intestinal metaplasia. Female gender was significantly associated with chronic active gastritis. *H. pylori* infection was associated with follicular gastritis, lymphoid aggregates, intestinal metaplasia, GIST and chronic active gastritis. The association between female gender and chronic active gastritis; and the association between H. pylori infection and GIST are both novel findings that have not been previously reported in the published literature. We are also in agreement with others [6] that histopathologic examination of LSG specimens may not be routinely needed and should be undertaken on selective basis, subject to the patients’ clinical picture or suspicious lesions detected intraoperatively. This study strengthens the thin evidence base required for the generation of solid guidelines in relation to the value of routine histopathologic examination of LSG specimens. The clinical relevance of the current research is that it confirmed that such activity, with its associated costs and efforts, might not be warranted.
